# Renovation Effect of Flax FRP-Reinforced Cracked Concrete Slabs under Impact Loadings

**DOI:** 10.3390/ma14206212

**Published:** 2021-10-19

**Authors:** Wenjie Wang, Zonglai Mo, Nawawi Chouw, Krishnan Jayaraman

**Affiliations:** 1 Jiangsu Key Laboratory of Engineering Mechanics, School of Civil Engineering, Southeast University, Nanjing 211189, China; 2 School of Mechanical Engineering, Nanjing University of Science and Technology, Nanjing 210094, China; mozonglai2000@163.com; 3 Department of Civil and Environmental Engineering, University of Auckland, Private Bag 92019, Auckland 1142, New Zealand; n.chouw@auckland.ac.nz; 4 Department of Mechanical Engineering, University of Auckland, Private Bag 92019, Auckland 1142, New Zealand; k.jayaraman@auckland.ac.nz

**Keywords:** drop weight impact, fibre-reinforced concrete slab, flax fibre-reinforced polymer, renovation method

## Abstract

The impact behaviour of flax fibre-reinforced polymer (FFRP) renovated coconut fibre-reinforced concrete (CFRC) slabs was investigated through two series of experiments and theoretical analysis. The first experiment was carried out to find out the effectiveness of FFRP retrofitted method for the partly damaged concrete structure and its performance under impact loadings. The renovation process was applied on the pre-cracked rectangular CFRC slabs of 600 mm × 300 mm × 50 mm with FFRP laminates, before the repeated impact tests. Then, the parameters of these slabs, i.e., impact force history, deflection history and damage pattern, were discussed in detail. Another experiment was conducted on the FFRP-CFRC square slabs with a dimension of 600 mm × 600 mm × 50 mm. Based on test results, the effect of different FFRP configurations was discussed to find out the effective reinforcement method. In addition, the two-degree-of-freedom spring-mass model was applied to predict the impact force. Results demonstrate that FFRP composites have a good potential to be utilised as renovated construction materials under dynamic load conditions.

## 1. Introduction

Fibre-reinforced polymers (FRP) were first presented in the 1940s, followed by various FRP reinforcing products invented in Europe and Asia in the 1970s and 1980s [1]. In the past two decades, fibre-reinforced polymer (FRP) composite materials have been widely industrialized as they are economical and structurally workable, serving as load-bearing elements in buildings and bridges.

Retrofitting is an art of structural modification, which becomes a more cost-effective superior alternative to the traditional techniques. In the field of concrete constructions, the FRP retrofitting method is able to provide improvement of the static and dynamic performance of infrastructures [2,3,4,5]. Mousavi and Shafei [2] investigated the impact resistance of hybrid FRP-steel-reinforced concrete slabs, results of which showed that FRP material reinforcement can minimize the damage level and dissipate the imposed energy. Abdel-Kader and Fouda [3] experimentally compared the performance between plain concrete and glass fibre-reinforced polymer (GFRP) sheet-strengthened concrete plates in terms of impact resistance under various compressive strengths. The results indicated that the GFRP reinforcement had a better performance, with a nonlinear ratio of improvement under different compressive strengths.

Blast response of one-way reinforced concrete slabs retrofitted with fibre-reinforced plastic was studied by Ahmad and Azar [6], where the influence of geometrical parameters, i.e., the number of layers and the fibre orientation, on the blast resistant was discussed. Kong et al. [7] investigated the blast resistance of aramid fibre-reinforced plastic (AFRP) sheet retrofitted concrete slabs by analysing the effects of AFRP layer, FRP type, strengthening mode, FRP bond strength, and TNT mass. The results indicated that AFRP composite displayed promising properties as an excellent blast-resistant strengthening material.

Natural fibre-reinforced polymer composite is developing rapidly lately in terms of its industrial applications, fundamental research, due to its low cost, low energy inputs, comparable mechanical properties, high specific strength, nonabrasive, and eco-friendliness [8,9,10,11]. In the field of bio-engineering application, the study touches on various aspects of FRP such as its biocompatibility advantages and critical analysis in an informative way [12]. Coir, jute, bagasse, cotton, bamboo, and hemp are used as natural fibres to reinforce polymer composites, which are eco-friendly, lightweight, strong, renewable, cheap and biodegradable, and can be used to reinforce both thermosetting and thermoplastic matrices. Thermosetting resins such as epoxy, polyester, polyurethane, phenolic are commonly used to reinforce composites for higher performance applications [13].

Flax fibre-reinforced polymer wrapped coconut fibre-reinforced concrete (FFRP-CFRC) composites have been experimentally studied in recent years. Yan and Chouw [14] carried out research on the properties of FFRP tube-encased concrete composites, and pointed out that FFRP substantially increased the strength of the composites. Wang and Chouw [15,16,17] carried out preliminary experimental research on the impact behaviour of the CFRC and FFRP-CFRC composites. However, the study on the retrofitting potential of FFRP composite has not been reported to the authors’ knowledge.

FRP composites renovation technology has been considered as an important method in civil construction engineering for retrofitting partly damaged structural components. Carbon fibre-reinforced polymer (CFRP), as an example, has been applied in buildings and bridges. In this study, the impact behaviour of FFRP-retrofitted CFRC slabs was investigated through experimental and analytical studies. Firstly, six CFRC rectangular slabs were slightly cracked using small impact energy, resulting in similar small damage to the slabs. Secondly, two FFRP configuration methods were applied to renovate these crack slabs. In the next stage, the repeat impact tests were conducted on these renovated FFRP-CFRC slabs, and the effectiveness of the two types of FFRP configurations was evaluated. A more effective wrapping configuration among different wrapping designs of the FFRP-strengthened CFRC slab were decided via impact test results, where their parameters were discussed in terms of impact force history, deflection history, energy absorption and damage pattern. The theoretical analysis method was also employed to predict the maximum impact force and the maximum deflection, and the results were compared with experimental results.

## 2. Experimental Program

### 2.1. Materials

- Cement, fine aggregate and coarse aggregate

Ordinary Portland cement sourced from Golden Bay Cement, New Zealand was adopted with relative density of 3.11 t/m^3^. Locally available sea sand passing through a 2.36 mm sieve was used. The fineness modulus of the sand was found to be 2.75. Crushed aggregate available from local sources has been used, with a diameter range of 7–13 mm and a fineness modulus of 3.48. It is mentioned that the above material parameters were provided by Winstone Aggregates, Auckland, New Zealand, who has conducted the test according to standard NZ 3111:1986 [18].

**Figure 1:**
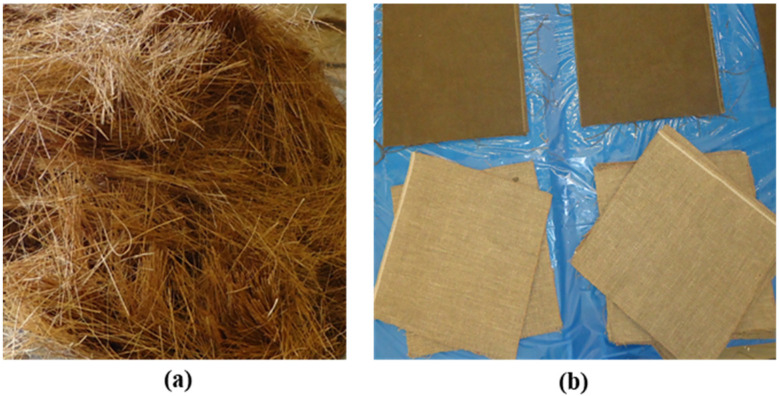
(**a**) Coconut fibre; (**b**) Flax fabric.

The coconut fibre was imported from Dewataru, Bali, Indonesia, the diameter and length of which are around 0.25 mm and 50 mm (Figure 1a), respectively, with a density of about 1.2 g/cm^3^ and 48-h water absorption of 180% [17]. Fibre preparation is necessary for obtaining the intended length of fibres and removing dust from the fibres. The details of the preparation can be found in our previous work [17]. The flax fabric (Figure 1b) was sourced from Lineo Company in Belgium. The flax fibre yarns have a density of 1.43 g/cm^3^, with a tensile strength of 145 MPa and tensile modulus of 16 GPa [19]. The flax fabric was handed laid-up with the adhesion of SP High Modulus PRIME^TM^ 20LV resin system, which was specifically designed for use in a variety of resin infusion processes including RTM (resin transfer moulding). The properties of the used epoxy system are displayed in Table 1, which was provided by Gurit, Auckland in New Zealand.

### 2.2. Specimens Preparation

Two experiments were carried out in the study. The concrete composite sample preparation is introduced, respectively, as follows.

#### 2.2.1. Specimens Preparation for Experiment I

Six rectangular CFRC slabs with a dimension of 600 mm × 300 mm × 50 mm were prepared for test I. These specimens were pre-cracked to simulate the impact-induced damage, generated by the same level of impact loading. The FFRP composites were then adhered to the cracked specimens for their renovation.

- Casting of CFRC slabs

The slab specimens were cast to the required size by using wood moulds, where two frames with a depth of 50 mm were connected to a flat plate with a dimension of 600 mm × 300 mm to form an open container.

Initially, the wood mould was coated with engine oil so that the slab specimens can be demoulded from it. Secondly, CFRC slabs were cast using a concrete mixer. The mix ratio for plain concrete (PC) is 1:0.48:2:2 for cement, water, sand, aggregate, respectively, and CFRC was designed the same as that of PC, except that the coconut fibres were added to the mixture, with the same amount of aggregate by mass being deducted from the total weight of the aggregate. The drinkable water was applied as the mixing water according to standard NZ 3121:2015 [20]. The coconut volume was 1.2% for CFRC. The detailed specimen casting procedures are listed in our previous work [16]. Table 2 lists the detailed mix design of the concrete composition.

The slump test was then performed and the results for CFRC were about 38 mm. The specimens were demoulded after 24 h and cured for 28 days in a fog room for proper temperature and humidity.

- Pre-cracking of CFRC slabs

**Figure 2:**
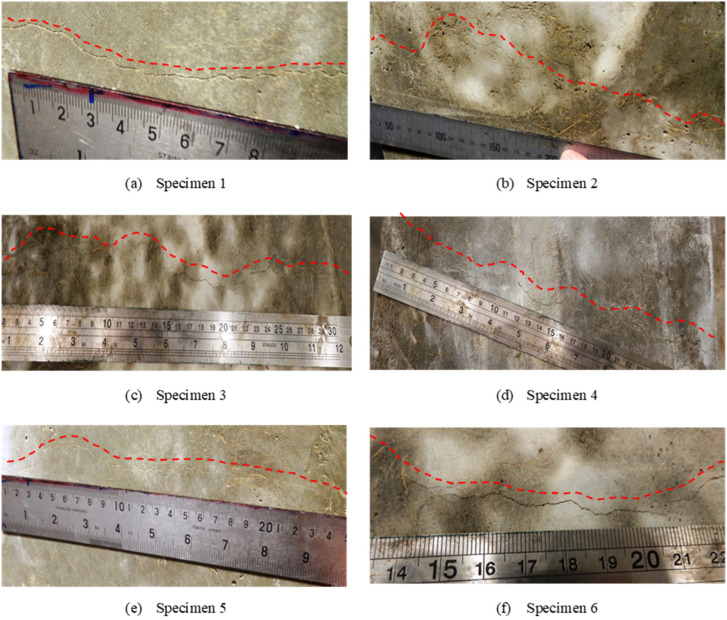
Crack damage of CFRC slabs under impact load.

All six specimens were cracked under the same drop weight and height, i.e., a drop weight of 40 kg and an impact height of 5 mm, to make sure all specimens had similar crack damage. The crack pattern of these six specimens was similar, as shown in Figure 2, indicating that similar damage was achieved. The crack occurred at the tensile side of the slab, located close to the specimen centre line, and the width was less than 0.5 mm.

**Figure 3:**
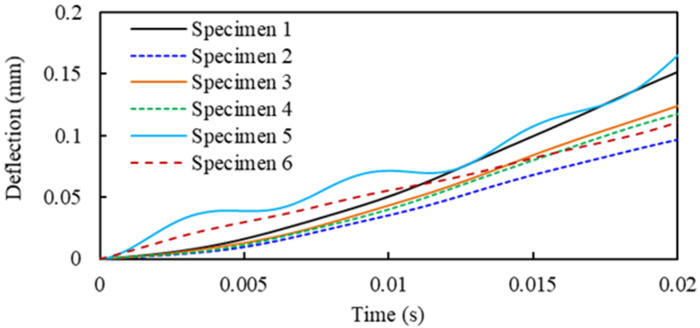
Deflection of CFRC slabs under an impact load.

The deflection histories of the specimens during the impact are displayed in Figure 3. It shows that specimens have a similar response under the same level of impact, but with slightly different values of deflection, which is due to the mixture effect of concrete where the fibre diffusion cannot be exactly controlled. The maximum deflection ranged from 0.89 mm to 1.65 mm.

- Fabrication of FFRP-CFRC slabs

**Figure 4:**
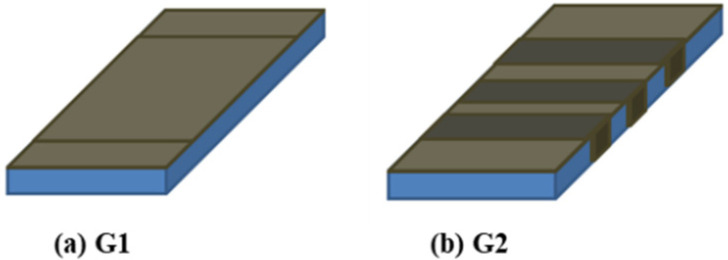
Sketches of renovated FFRP-CFRC slabs: (**a**) G1 and (**b**) G2.

FFRP laminates with a thickness of 3 mm were prepared and wrapped over the cracked CFRC slabs. Two configurations of FFRP-CFRC slabs in Figure 4 were studied with the same quantity of flax fabric of 0.5 m^2^ applied on each slab specimen. Specimens 1–3 were renovated by adhering FFRP laminates to the tensile surface, which is series G1, and specimens 4–6 were renovated by adhering both FFRP laminates and U-shape strips, which is series G2. The sketches of renovated FFRP-CFRC slabs are displayed in detail in Figure 4.

The fabrication procedure is described as follows: after cutting flax fabric, the epoxy system was mixed thoroughly for three minutes, then impregnating the flax fabric with the epoxy using a brush and cleaning the CFRC slab surface well to remove the dust. The next step entailed wrapping the epoxy-impregnated fabric tightly to the cracked CFRC slabs and curing the specimens at room temperature for at least 48 h.

#### 2.2.2. Specimens Preparation for Experiment II

Four square CFRC slabs (600 mm × 600 mm × 50 mm) with different FFRP laminates of reinforcement were prepared for test II. The influence of FFRP reinforcement configuration on the impact performance was investigated. The experiment was also a preliminary application test for FFRP composite in civil construction engineering.

**Figure 5:**
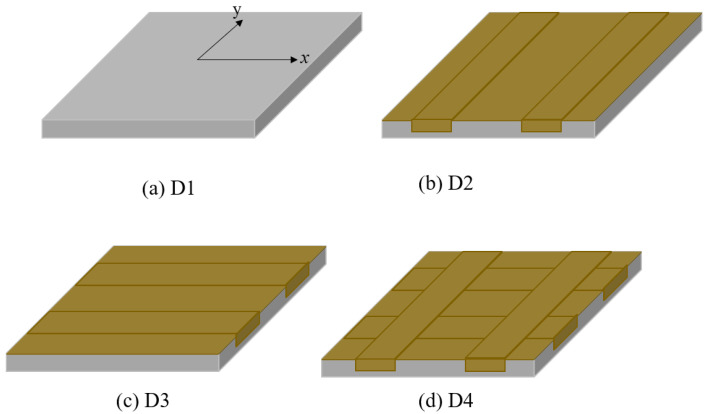
Sketches of renovated FFRP-CFRC square slabs.

The CFRC specimen casting procedure is the same as described in Section 2.2.1. After curing 28 days of CFRC, FFRP laminates with an area of 1.5 m^2^ were prepared and adhered to the CFRC square slabs. The FFRP-CFRC configurations are shown in Figure 5. D1, D2, D3, and D4 are four different renovation approaches. D1 means the specimen of CFRC as reference. D2 means FFRP laminates were attached to the tensile side of the slabs and two strips were attached along the X-axis. D3 means FFRP laminates were attached to the tensile side of cracked slabs and two strips were attached along the Y-axis. D3 means FFRP laminates were attached to the tensile side of the slabs and four FFRP strips were attached to both the X- and Y-axis.

### 2.3. Experimental Set-Up

#### 2.3.1. Impact Test Set-Up for Experiment I

Impact tests were carried out using a drop weight test rig, with variable impact heights (maximum heights of 2600 mm). The impact mass can be set from 30 kg to 200 kg, with an increment of 10 kg. The PCB dynamic load cell was monitored to record the impact force. The accelerometer provided by PCB Company of USA was employed to record the acceleration of the specimens during impacts. The laser displacement sensor sourced from Panasonic, Ōsaka, Japan was installed beneath the slab centre to measure the net deflection of the slab, and its data acquisition was set to 50 kHz.

**Figure 6:**
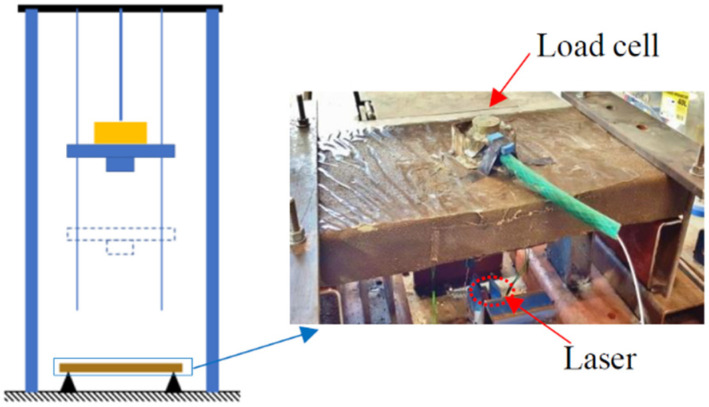
Sketch of impact machine and specimen set-up.

Repeat impact tests were carried out on G1 and G2 FFRP-CFRC slabs. The impact height was initially set to 10 mm, then increased to 30 mm, 50 mm, 70 mm, 100 mm, 150 mm, 200 mm, 250 mm for each subsequent strike until the specimen broke. The dynamic load cell and laser sensor were applied to measure the impact force and the deflection, respectively. Besides, strain gauges were attached to the centre of the slab tensile surface to measure the strain of FFRP laminates. A number of impact tests were conducted with increasing impact heights until a specimen was broken. The detail of the test set-up for the impact test is described in Figure 6. Table 3 lists the test matrix of the specimens.

#### 2.3.2. Impact Test Set-Up for Experiment II

**Figure 7:**
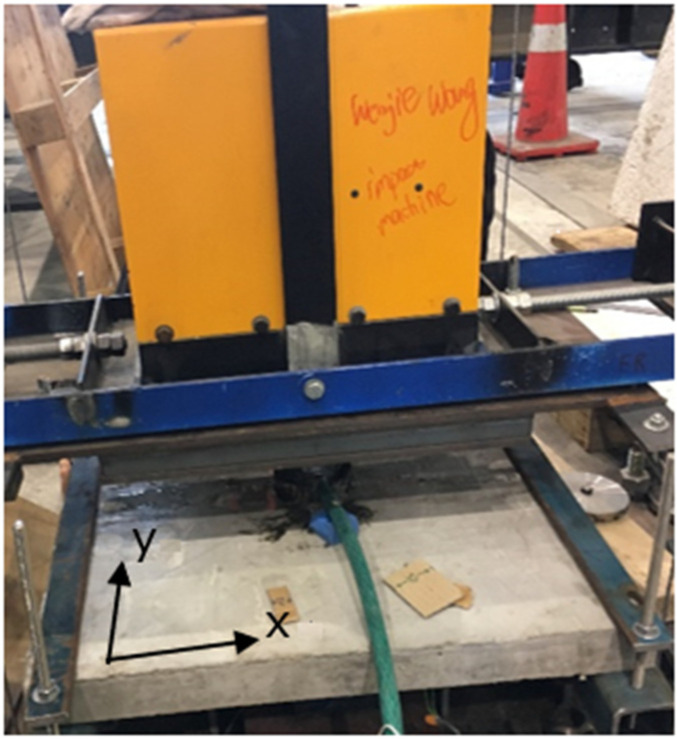
Test set-up for square specimen Group D.

Impact tests are similar as described above. Repeat impact tests were carried out on the FFRP-CFRC square specimens D1, D2, D3, and D4, respectively. The impact height was initially set to 10 mm, which then increased, then increased to 30 mm, 50 mm, 70 mm, 100 mm, 150 mm, 200 mm, 300 mm, 400 mm, 500 mm, 600 mm for each subsequent strike until the specimen broke. The test set-up for the impact test is described in Figure 7. In the test, the dynamic load cell was removed when the impact height was higher than 400 mm, as the specimen occurred a significant sag at the local central area. Hence, only the impact force within 400 mm of height was recorded. As for the strain, the record was continual until the final rupture of the specimen. The detailed test matrix can be seen in Table 3.

## 3. Experiment Results and Discussion

### 3.1. Experiment I

#### 3.1.1. Impact Force Time Histories

**Figure 8:**
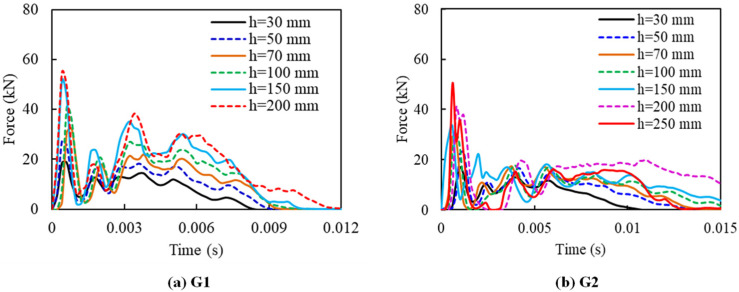
Impact force-time histories of G1 and G2.

Figure 8 displays the impact force histories of G1 and G2. The entire impact force-time period for slabs G1 and G2 are about 0.01 s and 0.015 s, respectively. The maximum impact force occurs at the very first stage of impact before 0.002 s. Multiple smaller force peaks were observed, which were called “secondary peaks” in the force history. This phenomenon can be explained by multiple contacts between the specimen surface and the impactor during the impact.

With regard to the total number of impacts required for failure, slab G1 suffered 6 blows of impact and slab G2 was fractured at the 7th impact at 250 mm of drop height. The maximum impact force increased with impact height. The maximum impact forces of G1 and G2 slabs were found to be about 54.79 kN and 50.26 kN, respectively. The results of series G1 and G2 were compared with the result of bare CFRC slabs to demonstrate the effectiveness of FFRP renovation in this study. The force-time history, deflection-time history and damage pattern of bare CFRC slabs can be found in the previous study [18]. The CFRC slab had a maximum impact force of about 6 kN, and the peak impact force occurred before 0.005 s. Two strikes took place for damaging the CFRC specimen with a failure height of 20 mm. It can be clearly found that flax fibre can greatly reinforce the impact resistance of pre-cracked CFRC, with the maximum impact force changed from 6 kN to over 50 kN.

#### 3.1.2. Deflection and Damage Development

**Figure 9:**
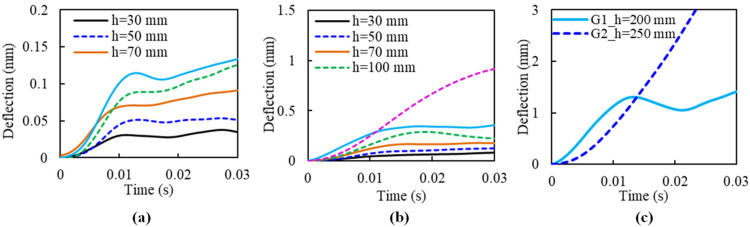
Deflection-time histories of G1 and G2 specimens under impact: (**a**) G1 with the impact height from 30 mm to 150 mm; (**b**) G2 with the impact height from 30 mm to 150 mm; (**c**) Deflection of G1 and G2 at failure.

Figure 9 displays the deflection of G1 and G2 during the impact, of which Figure 9a,b shows the undamaged situations, and Figure 9c provides the damage case (at the final impact with specimen fracture). The maximum deflection increased with the drop height. For slab G1, the maximum value increased from about 0.03 mm (30 mm of impact height) to 0.13 mm (150 mm of impact height). The deflection increases significantly at the last impact (200 mm of impact height), with a maximum value of about 1.5 mm. Slab G2 had a similar development of maximum deflection, while the values were larger than that of slab G1. For the same impact-loading situation, the maximum deflection of slab G2 increases from 0.07 mm to 0.35 mm, and the fracture deflection was about 3 mm, with a drop height of 250 mm.

**Figure 10:**
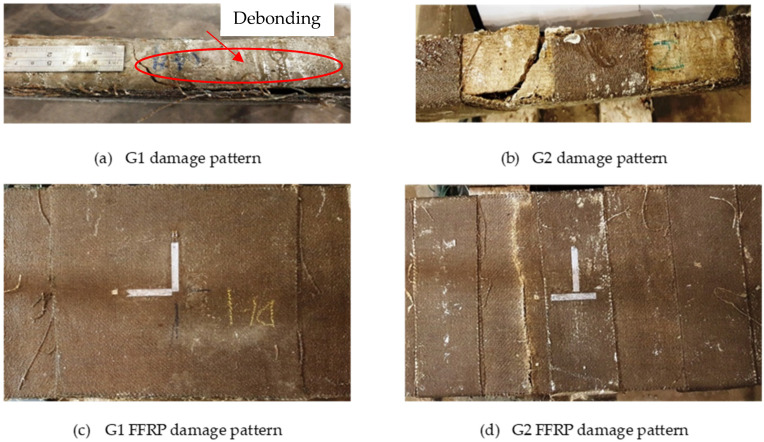
Damage patterns: (**a**) side view of G1 slab; (**b**) side view of G2 slab; (**c**) top view of G1 slab, and (**d**) top view of G2 slab.

**Figure 11:**
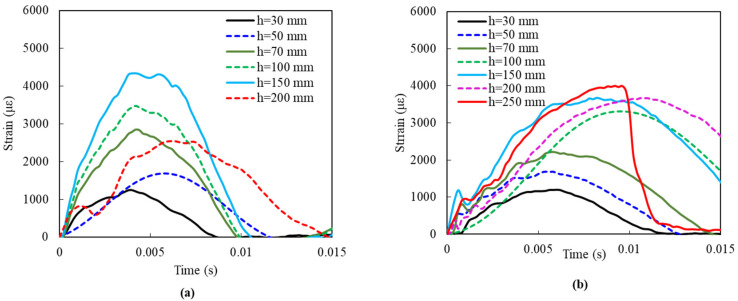
FFRP strain of (**a**) G1 and (**b**) G2 slabs.

The deflection difference between G1 and G2 could be due to their difference in damage patterns. Figure 10 shows their damage patterns. It is found that G1 occurred debonding failure when the drop height was 200 mm, as shown in Figure 10a, hence the test was stopped after this event. Whereas, slab G2 lasted until the fracture of FFRP laminates occurred, as shown in Figure 10d. This phenomenon can also be observed through the strain changes of FFRP laminates, shown in Figure 11. The strain in the case of G1 decreased suddenly to 2500 με at the 200 mm height impact, due to the FFRP laminates being debonded from the concrete core. On the other hand, FFRP strain in slab G2 increased gradually with the increment of drop height until the damage occurred.

Comparing the above renovation methods corresponding to slabs G1 and G2, respectively, it can be concluded that the G2 renovation method performed better than that of G1. It is also found that the G1 series was damaged due to debonding of FFRP laminate, leading to less ability in impact resistance. The FFRP laminate combined with its u-strip, such as the G2 series, could be an effective approach to retrofit the structural components. It is recommended that the debonding problem should be considered during the FFRP renovation design.

### 3.2. Experiment II

#### 3.2.1. Impact Force Time Histories

**Figure 12:**
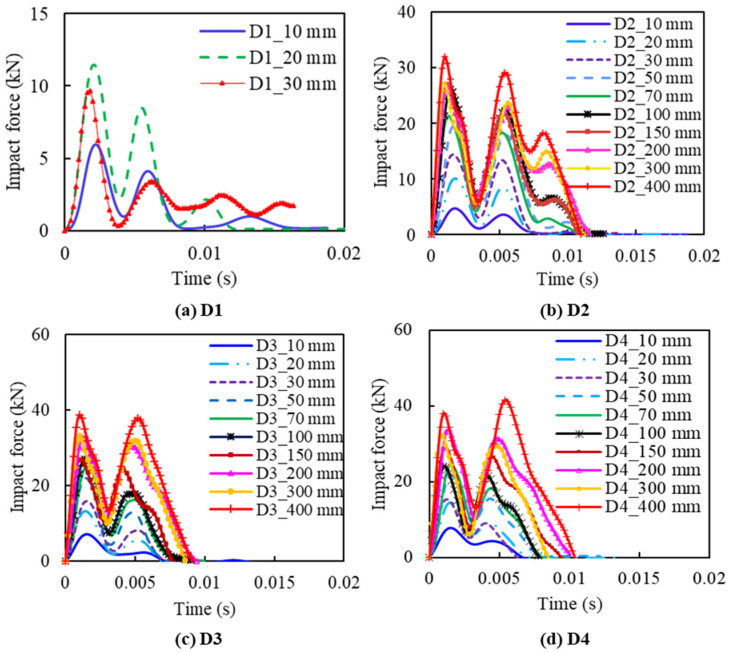
Impact force histories under different impact height for Group D: (**a**) specimen D1; (**b**) specimen D2; (**c**) specimen D3; (**d**) specimen D4.

Figure 12 shows the impact force histories of slabs D1, D2, D3, and D4. As a reference, the peak impact force of slab D1 was around 6 kN, 12 kN, and 10 kN, with impact heights of 10 mm, 20 mm, and 30 mm, respectively (shown in Figure 12a). The decrease of impact force at the last impact is due to the sudden rupture of the specimen, resulting in a loss of load-bearing capacity.

From Figure 12, a similar phenomenon, “secondary peaks”, as described, can also be observed, while the first two peaks had a very close value for the specimen series D2, D3, and D4, except for bare CFRC specimen D1. This could be due to the increase of stiffness of D2, D3, and D4 by FFRP reinforcement. The FFRP reinforcement greatly increases the elasticity of the specimen, resulting in an obvious rebounding phenomenon during impact. The results of slabs D2 show that the amplitude can go up to over 30 kN as the impact height increases to 400 mm, as shown in Figure 12b, while the slabs of D3 and D4, with a similar impact height of D2, tend to achieve higher impact forces. For example, slabs D3 and D4, with an impact height of 400 mm (Figure 12c,d), have a peak force of about 40 kN, which is 10 kN, outnumbering that of slabs D2. It is also notable that the response time length of slabs shows little connection with slab thicknesses, around 0.01 s for all slabs, with two main amplitude peaks appearing at around 0.002 s and 0.0054 s, respectively. Reinforced by FFRP laminate, the CFRC specimen can sustain higher capacity under repeat impact loadings. Among the four configurations of D1, D2, D3, and D4, the specimens D3 and D4 have the best impact resistance.

#### 3.2.2. Strain Time History

**Figure 13:**
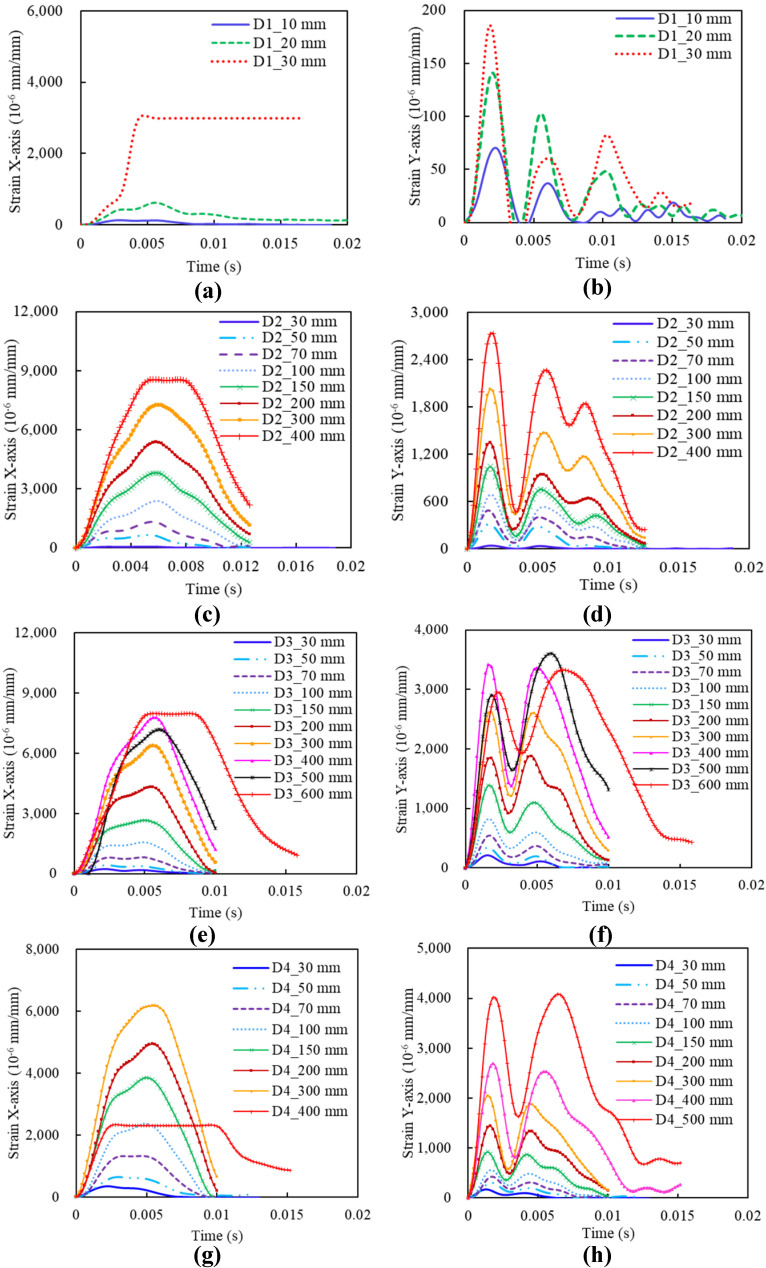
Strain time histories under different impact height for Group D: (**a**) X-axis strain of specimen D1; (**b**) Y-axis strain of specimen D1; (**c**) X-axis strain of specimen D2; (**d**) Y-axis strain of specimen D2; (**e**) X-axis strain of specimen D3; (**f**) Y-axis strain of specimen D3; (**g**) X-axis strain of specimen D4; (**h**) Y-axis strain of specimen D4.

Figure 13 shows the strain time histories of slabs D1, D2, D3, and D4 along the X- and Y-axis. A clear tendency is observed that, for the same type of slab, strain increases with the impact height, and the strain along the X-axis is more stable, with a higher value than that along the Y-axis. For different types of the slab, slabs D2 tend to achieve higher strain along the X-axis, while slabs D3 are higher in strain along the Y-axis. In addition, the time to the peak strain along the Y-axis shows an agreement with that of peak values of impact force in Figure 12, and the peak value along the X-axis is around 0.005 s. However, the strain along the X-axis performs different from the Y-axis, with only one fluctuation within 0.02 s, instead of two to three fluctuations along the Y-axis, which could be explained by the following reason. The specimen is fixed on the two edges at the two ends of the X-axis, leading to the constraining of the fluctuations at the X-axis during the impact. On the other hand, the fluctuations at the Y-axis are free to be generated as no constraints are applied.

For slabs D1, the strain along the Y-axis is nearly linear with thickness change, while the strain of D1_30 mm along the X-axis is way larger than those with other impact heights, as shown in Figure 13a. This is because the specimen occurred brittle fracture at the second impact with a height of 30 mm and the strain gauge along the X-axis was broken. In addition, it is notable that, after increasing to 300 mm of impact height for slabs D3, the increase of impact height leads to strain decrease, as shown in Figure 13e,f. A similar phenomenon is observed in slabs D4 in Figure 13g, where the strain of D4_400 mm shows a significant drop, from 6175 × 10^−6^ mm/mm of D4_300 mm to 2331 × 10^−6^ mm/mm of D4_400 mm. It means that the slab occurred a sudden fracture, giving rise to a rapid drawdown of the strain with the strain gauge was workable.

**Figure 14:**
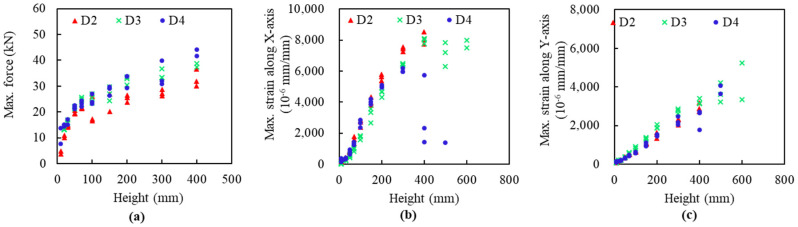
Comparison among D1, D2, and D3 for peak values under different impact heights: (**a**) Maximum forces, (**b**) Maximum strain along X-axis, (**c**) Maximum strain along Y-axis.

The detailed maximum values are compared from Figure 14. The slabs D4 have larger impact forces under the same impact height, while slabs D2 and slabs D3 have a slightly bigger strain along the X-axis and Y-axis, respectively. Comparing D2, D3, and D4, specimen D4 fractured at the height of 600 mm, followed by D3 with the height of 500 mm and D2 with 400 mm. This demonstrates that slab type D3 has the highest impact-resistant performance, indicating the best of the D3 FFRP configuration.

#### 3.2.3. Analytical Modelling

**Figure 15:**
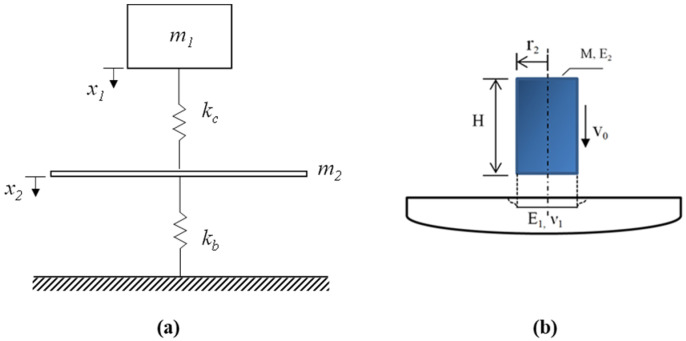
(**a**) Spring-mass model and (**b**) cylindrical striker impacting an elastic surface.

The studied impact results from the collision of two bodies, one with initial speed hitting another being at rest. This problem could be reduced to a two-degree-freedom of spring-mass system [21,22], which is composited of striking masse m_1_ and square slab mass m_2_, a contact spring with a stiffness *k_c_*, and another spring with a stiffness *k_b_*. The idealization of the spring-mass system is given in Figure 15a.

In this system, the striker and the square slab are connected by a spring representing Hertzian contact stiffness. The shear and the membrane stiffness are neglected in the present study. The combination of the springs reduces to a single spring representing the bending stiffness of the concrete slab. The contact stiffness for a slab impacted by a cylindrical impactor is given by the following expression [23,24]:

(1) $$\frac{1}{k_{c}}=\frac{1}{2r_{2}E_{1}}(1-\nu_{1}{}^{2}+\frac{E_{1}H}{\pi E_{2}r_{2}})$$

where $\nu_{1}$ is the Poisson ratio of the slab, $E_{1}$ is the Young modulus of the slab, *H* and *r*_2_ are the dimension of the striker, as shown in Figure 15b.

The procedure of determining the values of $\nu_{1}$ and $E_{1}$ were analysed in detail by the authors’ previous study [21]. The generalized bending stiffness of a square slab simply supported on two opposite edges is given by [24]:

(2) $$k_{b}=\frac{E_{1}h^{3}}{48(1-\nu_{1}^{2})}\frac{\pi^{4}}{L^{2}}$$

where *h* is the thickness of the slab and *L* is the length of the slab.

The dynamic equations of motion of the spring-mass model can be written as:

(3) $$\begin{array}{l} m_{1}{\ddot{x}}_{1}(t)+k_{c}[x_{1}(t)-x_{2}(t)]=0\\ m_{2}{\ddot{x}}_{2}(t)-k_{c}[x_{1}(t)-x_{2}(t)]+k_{b}x_{2}(t)=0 \end{array}$$

where *x*_1_ and *x*_2_ are the displacement of the striker and the slab, respectively.

The initial conditions of the system are given by:

(4) $$x_{1}(0)=0,\;{\dot{x}}_{1}(0)=0,\;x_{2}(0)=0,\;{\dot{x}}_{2}(0)=0$$

The coupled nonlinear differential equation is then solved using the Runge–Kutta method.

**Figure 16:**
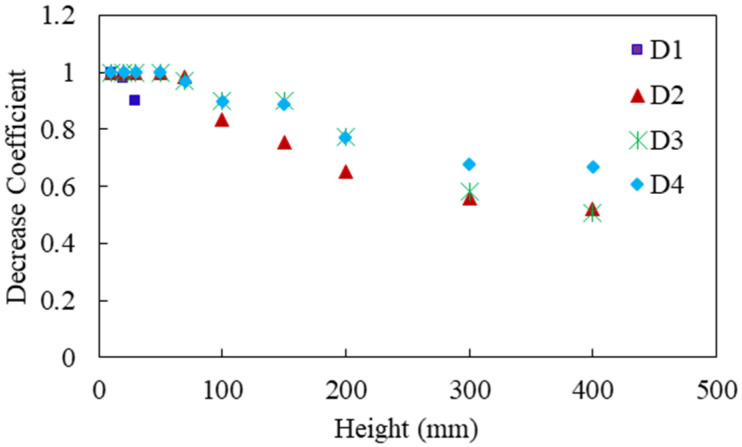
Estimated decrease coefficients of D1, D2, D3, and D4 slabs.

For the repeated impact, the specimen stiffness decreases due to the increase of the impact. To estimate the decrease of the stiffness in each impact, a similar method was applied as in the authors’ previous work [21], where the way of obtaining the decreasing coefficient was described in detail. Figure 16 displays the estimated decrease coefficients of D1, D2, D3, and D4 slabs.

**Figure 17:**
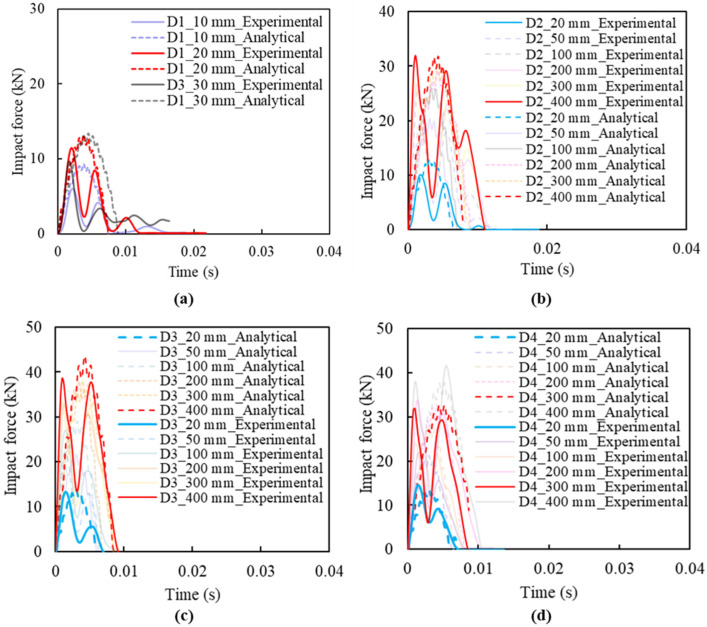
Impact force comparison of slabs between analytical results and experimental results: (**a**) Slab D1; (**b**) Slab D2; (**c**) Slab D3; (**d**) Slab D4.

The analytical results of impact force histories based on the spring-mass model are compared with experimental results in Figure 17 for verification. The results reveal the magnitude of the peak impact force, but with a time shift due to the assumptions made during the analytical modelling. This also indicates that the decrease coefficients or specimen stiffness should be accounted for when the repeated impact tests are carried out.

## 4. Conclusions

In this study, two experiments were conducted to investigate the application potential of a retrofitting method by using FFRP in a dynamic field.

Two different FFRP renovating configurations of pre-cracked slabs, i.e., G1 and G2 types, were tested to investigate the renovation effect of flax fibre. Experimental results indicate that flax fibre can greatly reinforce the impact resistance of pre-cracked CFRC, with maximum impact force changed from 6 kN to over 50 kN. Additionally, comparing the above renovation methods, it is advised that the G2 renovation method performed better than that of G1. This recommended that the debonding problem should be avoided when using FFRP laminates as renovation. The FFRP laminates combined with its u-strip could be an effective approach to retrofit the structural components.

Different FFRP reinforcement configurations were compared to find out a better way to reinforce concrete composites. An analytical spring-mass model was applied to predict the impact force. From the results obtained in this investigation, it can be concluded that: the maximum impact force increased with the increment of the impact height, with impact time duration of about 0.015 s for D1 and D2, and 0.009 s for D3 and D4. Results also show that the first peak impact force occurred at about 0.0054 s for all types of specimens. Similar to impact force, strain increases with the impact height for the same type of slab. In addition, the strain along the X-axis is more stable and with higher amplitude than that along the Y-axis. The strain along the X-axis performs differently from that of the Y-axis, where the X-axis strain-time curves displayed only one fluctuation within 0.02 s, while two to three fluctuations occurred for the Y-axis strain-time curves. It was also found that the impact resistant behaviour of D3 and D4 performed better than that of D1 and D2 in terms of impact force history, strain time history and impact striker numbers that a specimen can hold.

## Figures and Tables

**Table 1:** Mechanical properties of epoxy system.

Properties	Epoxy System	
	20LV Resin	Slow Hardener
Elastic modulus (GPa)	3.5	
Tensile strength (MPa)	73	
Strain to failure (%)	3.5	
Cured density (g/cm^3^)	1.144	
Linear shrinkage (%)	1.765	
Barcol hardness	27	
Density (g/cm^3^)	1.123	0.936
Volume ratio	100	31.4
Viscosity @20 °C (cP)	1010–1070	22–24

**Table 2:** Mix design details of the specimens.

Specimen Type	Water (kg/m^3^)	Cement (kg/m^3^)	Coarse Aggregate (kg/m^3^)	Fine Aggregate (kg/m^3^)	Coconut Fibre (kg/m^3^)
CFRC	222.3	427.5	855	855	12.83

**Table 3:** Test matrix of the specimens.

Specimen Group	Experiment Group	No. of Specimens	Concrete Core Dimension	FFRP Area (m^2^)
CFRC		3	600 mm × 300 mm × 50 mm	-
G1	Experiment I	3	600 mm × 300 mm × 50 mm	0.5
G2		3	600 mm × 300 mm × 50 mm	0.5
D1	Experiment II	2	600 mm × 600 mm × 50 mm	-
D2		2	600 mm × 600 mm × 50 mm	1.5
D3		2	600 mm × 600 mm × 50 mm	1.5
D4		2	600 mm × 600 mm × 50 mm	1.5

## Data Availability

Part of the data underlying this article will be shared on reasonable request from the corresponding author.
